# Risk stratification for Pseudomonas LRTI in immunocompromised patients: A LASSO-logistic regression model with clinical implications

**DOI:** 10.1097/MD.0000000000043836

**Published:** 2025-08-15

**Authors:** Xiao Ge, Chengjun Gan, Xu Han, Ling Chen, Jiebai Zhou, Yahui Liu, Yun Feng, Haixing Zhu

**Affiliations:** a Department of Respiratory and Critical Care Medicine, Ruijin Hospital Affiliated Shanghai Jiao Tong University School of Medicine, Shanghai, China; b Institute of Respiratory Diseases, School of Medicine, Shanghai Jiao Tong University, Shanghai, China; c Department of Respiratory and Critical Care Medicine, Second People’s Hospital of Wuwei, Wuwei, China; d Department of Critical Care Medicine, First People’s Hospital of Lanzhou, Lanzhou, China; e Department of Pulmonary Medicine, Zhongshan Hospital, Fudan University, Shanghai, China.

**Keywords:** immunocompromised patients, LASSO-logistic regression, lower respiratory tract infection, prognostic biomarkers, *Pseudomonas aeruginosa*

## Abstract

This retrospective cohort study examined 28-day mortality predictors among 177 immunocompromised adults with acute *Pseudomonas aeruginosa* lower respiratory tract infections (LRTIs) over a 6-year observational period. LASSO regression followed by logistic regression analysis was used to screen 19 clinical and laboratory variables. To evaluate the predictive performance of the model, ROC analysis was performed. Six independent mortality predictors emerged: neutrophil count, D-dimer-to-lymphocyte ratio (DLR), admission aspartate aminotransferase (AST), age, red cell distribution width (RDW), and platelet-to-lymphocyte ratio (PLR). The predictive model demonstrated robust discrimination (AUC = 0.792) with 91.9% sensitivity and 89.5% specificity at the optimal cutoff. The neutrophil-DLR-AST triad emerged as a novel prognostic combination reflecting systemic inflammation, coagulopathy, and hepatic stress. This study identifies 6 clinically accessible biomarkers for mortality risk stratification in immunocompromised hosts with *P aeruginosa* LRTI, with the neutrophil-DLR-AST triad representing a new prognostic model. The developed model offers clinicians a practical tool for early high-risk patient identification and personalized management. While highlighting the interaction between inflammatory regulation, coagulation abnormalities, and organ dysfunction in determining outcomes, these findings require prospective validation in multicenter cohorts. Future research should elucidate the pathophysiological mechanisms linking these biomarkers to disease progression and explore targeted interventions based on individualized risk profiles.

## 1. Introduction

*Pseudomonas aeruginosa* (PA) lower respiratory tract infections (LRTIs) present a severe threat to immunocompromised individuals, with 28-day mortality rates surpassing 40% despite intensive antimicrobial treatment.^[1–3]^ Managing these infections in such patients remains difficult. Existing prognostic models, designed for general community-acquired pneumonia, often underperform for this vulnerable group, failing to account for the multiorgan dysfunction common in immunodeficiency.^[4,5]^ Additionally, the rise of multidrug-resistant (MDR) *P aeruginosa* strains during ICU stays complicates treatment.^[6,7]^ Our study on clinical indices offers new insights into prognostic indicators for immunocompromised patients with *P aeruginosa* LRTI.

Accumulating evidence identifies the potential of risk biomarkers in immunocompromised populations.^[8,9]^ The degree of immunosuppression is a key determinant of LRTI risk in these populations. Lymphocytopenia (absolute lymphocyte count < 100/mm^3^)^[10]^ and neutropenia^[11]^ have been identified as independent predictors of LRTI. Additionally, the D-dimer-to-lymphocyte ratio (DLR), reflecting thromboinflammatory crosstalk, and elevated aspartate aminotransferase (AST), signaling hepatic stress from systemic inflammation, have emerged as relevant biomarkers.^[12,13]^ While individual markers such as the platelet-to-lymphocyte ratio (PLR) show promise in community-acquired pneumonia, their performance is limited in *P aeruginosa*-specific models due to collinear variable interference and failure to address immunodeficiency-associated end-organ vulnerability.^[14,15]^ Crucially, a comprehensive risk scoring system for LRTI in immunosuppressed populations is currently lacking. Reliable risk predictions developed and validated by integrating multiple clinical risk indicators are needed.

This 7-year retrospective cohort study spanning from 2016 to 2022 involved 177 immunocompromised adults diagnosed with *P aeruginosa* (LRTIs). The study aimed to address existing gaps in knowledge through the utilization of Lasso regression for variable selection. Our findings revealed a clinically relevant biomarker set comprising neutrophil count, DLR, and AST, which outperformed traditional severity scores. Additionally, we identified that red cell distribution width (RDW) and (PLR) serve as cost-effective modifiers that improve risk stratification, particularly beneficial in settings with limited resources. Furthermore, we developed a model that achieved a sensitivity of 91.9% in detecting early mortality, surpassing the predictive capabilities of most existing tools. By systematically addressing collinearity issues among candidate variables, our approach establishes a validated framework for personalized management of *P aeruginosa* LRTI in the expanding population of immunocompromised individuals.

## 2. Materials and methods

### 2.1. Patients and definitions

This single-center retrospective analysis evaluated adults aged ≥ 18 years with lower respiratory tract cultures positive for *P aeruginosa* between January 2016 and June 2022. All clinical specimens were obtained through standard bronchoscopic sampling procedures during hospitalization. Exclusion criteria encompassed cases with polymicrobial (LRTIs). Bacteremia recurrence was defined as the emergence of new positive respiratory cultures ≥ 48 hours after documented resolution of prior antimicrobial therapy. For patients experiencing multiple infection episodes during the study window, only the index event was included in the final dataset. The study was approved by the Ruijin Hospital Ethics Committee, Shanghai Jiao Tong University School of Medicine.

Immunocompromised status involve various conditions, including receiving immunosuppressive therapy like corticosteroids (specifically, prednisone at a dose exceeding 20 mg daily for a minimum of 14 days), methotrexate, cyclosporine, azathioprine, or biological modifiers in the past 3 months; undergoing transplantation of solid organs, stem cells, or bone marrow; being diagnosed with cancer, including solid organ tumors and hematological malignancies; experiencing severe burns; having diabetes; having liver cirrhosis; or being in a critical postoperative state.

### 2.2. Data preprocessing and missing values handling

During the data preparation stage of this research involving 177 immunocompromised patients with acute *P aeruginosa* LRTI, the presence of missing values was noted. To ensure the integrity and reliability of the data for subsequent LASSO-logistic regression analysis, 2 main strategies for handling missing data were employed: case deletion and median imputation. In the case deletion approach, each patient record with missing values was carefully examined, and those with extensive missing data that could introduce bias or make the analysis impractical were excluded from the dataset, resulting in the removal of a specific number of cases. For the median imputation method, when continuous variables had missing entries, the median of the available values for each variable was calculated, and the missing values were replaced with these median values. This approach aimed to maintain the characteristics of the data distribution while minimizing the negative impact of missing values. As a result of these data cleaning procedures, the sample size was reduced from 177 to 164.

### 2.3. Statistical analysis

We employed the t-test for the evaluation of continuous variables and the χ2 test for categorical variables, utilizing SPSS Statistics for Macintosh version 25.0 (IBM Corp., Armonk). The LASSO-logistic regression model was implemented to pinpoint the most critical prognostic risk factors associated with acute *P aeruginosa* (LRTI). All analyses were performed using R software (version 4.1.3). The outcomes of the LASSO-logistic regression analysis were produced through the R software package “glmnet” via the CNSknowall platform (http://cnsknowall.com/index.html#/HomePage), which serves as a comprehensive web resource for the analysis and visualization of biomedical data. Our team of data analysis experts conducted all statistical evaluations.

During the data preparation stage of this research involving 177 immunocompromised patients with acute *P aeruginosa* LRTI, the presence of missing values posed a challenge. To guarantee the integrity and dependability of the data for further LASSO-logistic regression analysis, 2 primary data cleaning strategies were employed: case deletion and median imputation. In the case deletion approach, each patient record with missing values was meticulously scrutinized. Those cases with extensive missing data that could potentially introduce bias or render the analysis unfeasible were excluded from the dataset. This eliminated a specific number of cases from the process. For the median imputation method, when dealing with continuous variables that had missing entries, the median of the available values for each respective variable was computed. The median value for every variable was calculated accurately and then the missing data was filled with those median values. In consequence, the resulting data cleaning procedures managed to bring the sample size down from 177 to 164.

## 3. Results

**Figure 1.**
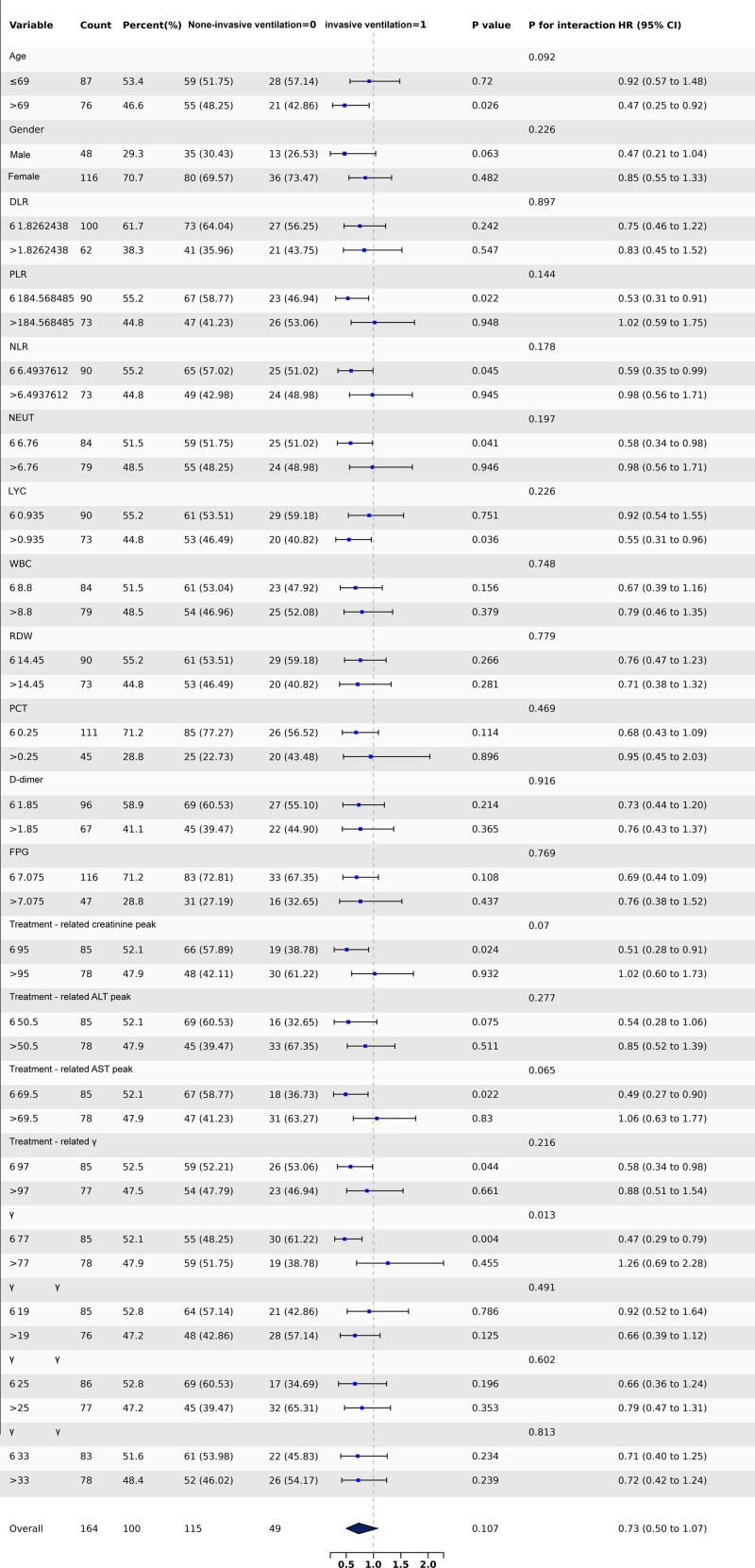
Primary illness characteristics of acute *P aeruginosa* lower respiratory tract infections in immunosuppressed patients.

In this study, we examined the population distribution characteristics and laboratory parameters in a cohort of 164 immunocompromised patients diagnosed with acute *P aeruginosa* (LRTIs). We investigated the impact of invasive ventilation on 28-day mortality with univariate analysis (Fig. 1). For patients aged 69 years or younger, the *P*-value was .72, with a hazard ratio (HR) of 0.92 (95% confidence interval [CI]: 0.57–1.48). In contrast, for patients older than 69, we observed a statistically significant *P*-value of .026, with an HR of 0.47 (95% CI: 0.25–0.92), suggesting that invasive ventilation may be associated with a lower mortality risk in this older group. The overall *P* value for gender is .949, indicating no significant difference in infection outcomes between female and male patients.

We conducted more analysis of hematological parameters before infection, focusing on white blood cell count (WBC), platelet count (PLT), peripheral blood lymphocyte count (LYC), and neutrophil count (NEUT), alongside coagulation and inflammatory markers such as D-dimer and procalcitonin (PCT). Additionally, we evaluated liver and kidney function indicators, examining both baseline values before treatment and peak values during treatment. Our analysis also included the neutrophil-to-lymphocyte ratio (NLR), (PLR), and derived lymphocyte ratio (DLR). Our previous research and other systematic reviews have underscored the importance of NLR in respiratory infections,^[14,15]^ and in this study, we aim to identify the key factors among these indicators to improve our understanding of their roles in clinical management and prognosis prediction. The results revealed that the DLR had a *P*-value of 0.226, indicating no significant association with the outcome. Also, for the NLR, levels at or below 6.49 produced a *P*-value of 0.045 and a HR of 0.59 (95% CI: 0.35–0.99), suggesting a potential interaction effect with mortality. However, when NLR levels surpassed 6.49, the *P*-value rose to 0.945, and the HR was 0.98 (95% CI: 0.56–1.71).

In conclusion, the univariate analysis revealed a complex interplay among these factors and the outcomes, emphasizing the need for advanced statistical techniques, such as LASSO-logistic regression, to accurately identify the key risk factors that significantly impact 28-day mortality in immunocompromised patients suffering from acute *P aeruginosa* (LRTIs).

### 3.1. LASSO regression analysis for identifying prognostic factors in acute P aeruginosa LRTI

**Figure 2.**
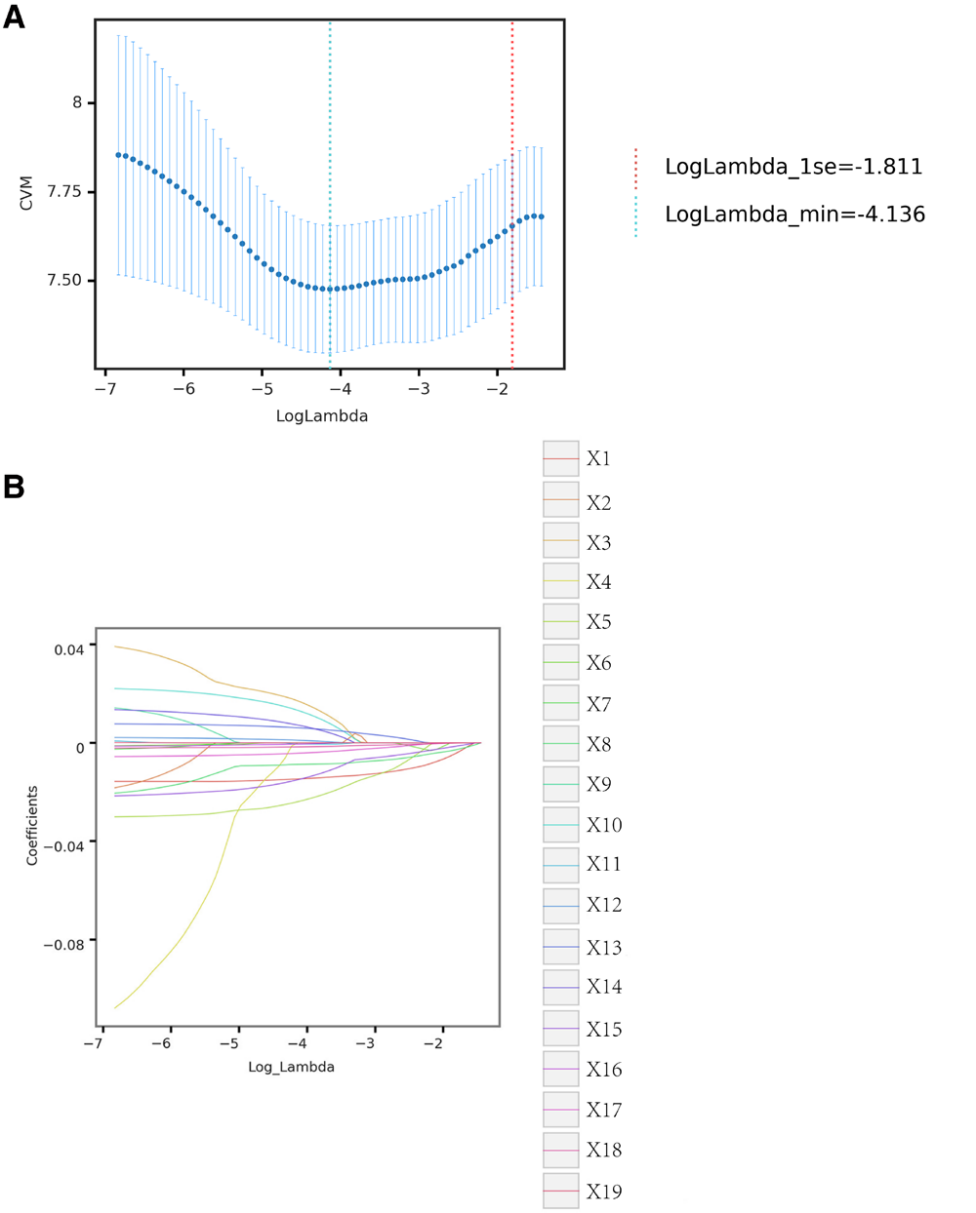
LASSO regression analysis for prognostic factors. (A) The cross-validation results for the LASSO regression model. The middle value between the 2 dotted lines represents the range of the positive and negative standard deviations of log(λ). The dotted line on the left indicates the value of the harmonic parameter log(λ) when the model error is minimized. Six variables were selected when log(λ) = −4.136. (B) LASSO coefficient profiles of the 19 variables. A vertical line was drawn at the value chosen by 10-fold cross-validation. As the value of λ decreased, the degree of model compression increased, and the function of the model to select important variables became more pronounced. The selected predictors include age, NEUT, DLR, baseline AST level prior to treatment, RDW, and PLR. The performance of the risk factor classifier, built using the LASSO-logistic regression model, is illustrated in the corresponding figures. The identified risk factors were systematically presented in the analysis, underscoring their importance in assessing the prognosis of this patient population. AST = aspartate aminotransferase, DLR = D-dimer-to-lymphocyte ratio, LASSO = least absolute shrinkage and selection operator, NEUT = neutrophil count, PLR = platelet-to-lymphocyte ratio, RDW = red cell distribution width.

This study utilized LASSO regression analysis to identify 6 optimal predictors from a total of 20 parameters that may influence the prognosis. The selected predictors include age, NEUT, DLR, baseline AST level before treatment, RDW, and PLR (Fig. 2; Table 1). Detailed characteristics of these risk factors are summarized in Supplemental Digital Content 1 (Table S1, Supplemental Digital Content, https://links.lww.com/MD/P630, for complete statistical analysis).

**Table 1:** Risk factors selected by LASSO-logistic regression model.

Variables	Risk factors
X1	Age
X2	WBC
X3	PLT
X4	NEUT (peripheral blood neutrophil count)
X5	LN
X6	NLR
X7	D-dimer-to-lymphocyte ratio (DLR)
X8	Platelet-to-lymphocyte ratio (PLR)
X9	Red cell distribution width (RDW)
X10	DD
X11	PCT
X12	Admission creatinine
X13	Maximum creatinine during treatment
X14	Admission ALT
X15	Maximum ALT during treatment
X16	Admission aspartate aminotransferase (AST)
X17	Maximum AST during treatment
X18	Admission γ-GTP
X19	Maximum γ-GTP during treatment

ALT = alanine aminotransferase, AST = aspartate aminotransferase, DD = D-dimer, GTP = glutamyl transpeptidase, LASSO = least absolute shrinkage and selection operator, NEUT = neutrophil count, NLR = neutrophil-to-lymphocyte ratio, PCT = procalcitonin, PLT = platelet count, WBC = white blood cell count.

To validate the LASSO-logistic model, a generalized cross-validation was performed. As the log(λ) value of the harmonic parameter varied, it was observed that the area under the curve (AUC) of the model also fluctuated. The performance of the risk factor classifier, built using the LASSO-logistic regression model, is illustrated in the corresponding figures. The identified risk factors were systematically presented in the analysis, underscoring their importance in assessing the prognosis of this patient population.

### 3.2. Lasso regression model performance evaluation

**Figure 3.**
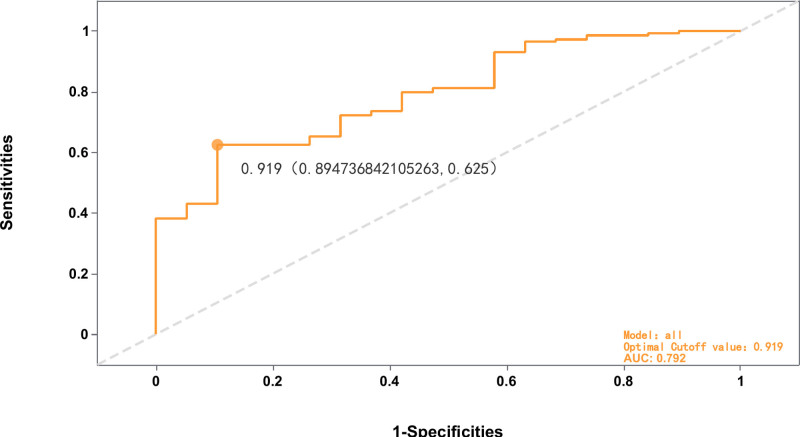
Joint ROC curve of the 6 Factors in the LASSO regression model. LASSO = least absolute shrinkage and selection operator, ROC = receiver operating characteristic.

The evaluation of the Lasso regression model’s performance revealed 6 significant prognostic factors. Following this identification, we validated the diagnostic performance metrics of the model (Fig. 3). To assess the model’s effectiveness at various thresholds, we analyzed the joint ROC curve for these 6 key factors, determining the optimal cutoff value to be 0.919. The model’s AUC value was calculated to be 0.792, indicating a reasonable level of predictive ability. The AUC of 0.792 in our model suggests it is effective in predicting risk factors and outcomes associated with acute *P aeruginosa* LRTI in immunocompromised patients. In terms of sensitivity and specificity, at the optimal cutoff of 0.919, the model achieved a sensitivity of 0.919 and a specificity of approximately 0.895 (specifically 0.894), with a false positive rate of 0.625.

## 4. Discussion

This study highlights the significance of the neutrophil-DLR-AST triad as a crucial prognostic indicator for mortality in immunocompromised patients with acute (LRTIs) caused by (*P aeruginosa*), reflecting *P aeruginosa*-specific pathophysiological mechanisms. The *P aeruginosa*-induced type III secretion system (T3SS) stimulates excessive neutrophil extracellular trap formation, leading to aggravated tissue damage.^[16]^ The unbalanced neutrophil in our study is consistent with previous evidence indicating that severe neutropenia (<1.0 × 10^9^/L) is a major risk factor for *P aeruginosa*-LRTI in immunocompromised individuals.^[17]^ Moreover, an elevated DLR indicates concurrent hypercoagulability and immune exhaustion, which correlates with the observed high mortality rate in *P aeruginosa* bacteremia.^[11]^ Lymphopenia further worsens outcomes, as evidenced in patients with hematologic malignancies.^[10]^ Additionally, *P aeruginosa* exotoxin A induces mitochondrial injury in hepatocytes, with studies linking hepatic dysfunction to poorer outcomes in LRTI.^[18]^ Consequently, this triad comprehensively represents systemic *P aeruginosa* toxicity by integrating inflammation, coagulation, and organ damage pathways.

Risk profiles for bacterial pneumonia in immunocompromised groups. The identification of neutropenia as a crucial risk factor confirms previous findings that *P aeruginosa* infections are significantly more prevalent among neutropenic individuals with hematologic malignancies.^[10]^ Notably, our use of LASSO-based variable selection addressed collinearity between neutrophil counts and lymphocyte ratios, representing a methodological advancement over conventional logistic regression models that may overlook interrelationships among inflammatory markers. Additionally, the association of hepatic dysfunction (as indicated by AST elevation) with mortality mirrors findings from cohort studies linking *P aeruginosa* exotoxin A-induced hepatocyte damage to impaired metabolic clearance and sepsis progression.^[19]^ This corresponds with reports suggesting that chronic pulmonary conditions (e.g., COPD, bronchiectasis) heighten the risk of *P aeruginosa*-LRTI by compromising mucociliary clearance,^[17]^ although our model prioritized acute-phase biomarkers over chronic ailments in predicting mortality. Interestingly, while previous research highlighted the prognostic significance of D-dimer in bacteremia,^[11]^ our incorporation of DLR as a composite measure captures both hypercoagulability and adaptive immune dysfunction—a novel integration supported by recent evidence of NETosis-driven microthrombosis in *P aeruginosa* pneumonia.^[20]^ univariate analysis revealed that while NLR, PLR, and DLR each demonstrated some predictive capacity, none proved sufficient as standalone prognostic markers.^[21]^ This finding is consistent with a 2022 ICU study of pneumonia patients, which found that although these hematologic indices offered some prognostic information, the APACHE IV score remained superior for mortality prediction.^[22]^ Our Lasso regression analysis further substantiated the need for a multifactorial approach. The integration of NLR, PLR, and DLR with other clinical and laboratory parameters significantly enhanced predictive performance. These results underscore the importance of incorporating these hematologic markers into comprehensive prognostic models rather than relying on them independently. These results collectively validate the pathobiological coherence of our triad and illustrate how Lasso regression-driven variable selection enhances predictive accuracy in diverse immunocompromised populations.

Nevertheless, the study is constrained by several limitations. Primarily, the small sample sizes and the single-center design may not be representative of larger urban populations. Furthermore, our results lack experimental validation to establish the reliability of our model. Future research efforts should focus on validating these findings using larger, multicenter cohorts and assessing the long-term impact of biomarkers on patient outcomes. These limitations will be addressed in future studies, ultimately enhancing our comprehension of the role of acute *P aeruginosa* LRTI in immunocompromised patients and improving clinical practices in such scenarios.

In conclusion, this study successfully identifies key prognostic factors associated with 28-day mortality in immunocompromised individuals with acute LRTIs attributed to *P aeruginosa*. Through the application of LASSO-logistic regression, 6 notable predictors have been identified, offering potential enhancements in risk evaluation and medical care. These results underscore the importance of integrating these prognostic factors into routine clinical protocols, potentially leading to enhanced patient outcomes via personalized interventions. Subsequent research efforts should focus on validating these predictors across broader and more diverse patient cohorts, as well as exploring their underlying mechanisms to strengthen clinical decision-making.

## Acknowledgments

We thank all participants for their invaluable contributions to this research.

## Author contributions

**Conceptualization:** Chengjun Gan, Ling Chen, Haixing Zhu.

**Data curation:** Xiao Ge, Chengjun Gan, Xu Han, Haixing Zhu.

**Formal analysis:** Xiao Ge, Chengjun Gan, Xu Han, Ling Chen, Haixing Zhu.

**Funding acquisition:** Ling Chen, Jiebai Zhou, Haixing Zhu.

**Investigation:** Xu Han.

**Methodology:** Chengjun Gan, Xu Han, Ling Chen, Yahui Liu, Yun Feng.

**Project administration:** Xu Han, Yahui Liu.

**Resources:** Yahui Liu.

**Supervision:** Jiebai Zhou, Yun Feng.

**Visualization:** Ling Chen, Haixing Zhu.

**Writing – original draft:** Xiao Ge, Haixing Zhu.

**Writing – review & editing:** Ling Chen, Jiebai Zhou, Yun Feng.

## Supplementary Material


