# Supplementary material for: Risk stratification for Pseudomonas LRTI in immunocompromised patients: A LASSO-logistic regression model with clinical implications
Source: Medicine (Baltimore). 2025 Aug 15;104(33):e43836. doi: 10.1097/MD.0000000000043836 (PMC12366886; doi:10.1097/MD.0000000000043836)
Supplement: Supplementary file 1 [file medi-104-e43836-s001.doc]

**Supplemental Digital Content**

Risk factors selected by LASSO-logistic regression model (Top 6 by absolute value of coeff_min_lamda).

| Variables | Risk factors | Coefficient |
| --- | --- | --- |
| X8 | Platelet to lymphocyte ratio (PLR) | 0.0088 |
| X9 | Red cell distribution width (RDW) | 0.0132 |
| X1 | Age | 0.0147 |
| X16 | Admission aspartate aminotransferase (AST) level before treatment | 0.015 |
| X7 | D-dimer to lymphocyte ratio (DLR) | 0.017 |
| X4 | NEUT (peripheral blood neutrophil count) | 0.0239 |
